# Lipid membrane templated misfolding and self-assembly of intrinsically disordered tau protein

**DOI:** 10.1038/s41598-020-70208-6

**Published:** 2020-08-07

**Authors:** Jaroslaw Majewski, Emmalee M. Jones, Crystal M. Vander Zanden, Jacek Biernat, Eckhard Mandelkow, Eva Y. Chi

**Affiliations:** 1 Division of Molecular and Cellular Biology, National Science Foundation, Alexandria, VA 22314 USA; 2 Nanoscience and Microsystems Engineering Graduate Program, University of New Mexico, Albuquerque, NM 87131 USA; 3 Department of Chemical and Biological Engineering and Center for Biomedical Engineering, University of New Mexico, Albuquerque, NM 87131 USA; 4 Department of Chemistry and Biochemistry, University of Colorado at Colorado Springs, Colorado Springs, CO 80918 USA; 5 Center for Neurodegenerative Diseases (DZNE), 53127 Bonn, Germany; 6 CAESAR Research Center, 53175 Bonn, Germany; 7 Theoretical Biology and Biophysics Division, Los Alamos National Laboratory, Los Alamos, NM 87545 USA

**Keywords:** Molecular biophysics, Supramolecular assembly, Intrinsically disordered proteins, Biophysics

## Abstract

The aggregation of the intrinsically disordered tau protein into highly ordered β-sheet-rich fibrils is implicated in the pathogenesis of a range of neurodegenerative disorders. The mechanism of tau fibrillogenesis remains unresolved, particularly early events that trigger the misfolding and assembly of the otherwise soluble and stable tau. We investigated the role the lipid membrane plays in modulating the aggregation of three tau variants, the largest isoform hTau40, the truncated construct K18, and a hyperphosphorylation-mimicking mutant hTau40/3Epi. Despite being charged and soluble, the tau proteins were also highly surface active and favorably interacted with anionic lipid monolayers at the air/water interface. Membrane binding of tau also led to the formation of a macroscopic, gelatinous layer at the air/water interface, possibly related to tau phase separation. At the molecular level, tau assembled into oligomers composed of ~ 40 proteins misfolded in a β-sheet conformation at the membrane surface, as detected by in situ synchrotron grazing-incidence X-ray diffraction. Concomitantly, membrane morphology and lipid packing became disrupted. Our findings support a general tau aggregation mechanism wherein tau’s inherent surface activity and favorable interactions with anionic lipids drive tau-membrane association, inducing misfolding and self-assembly of the disordered tau into β-sheet-rich oligomers that subsequently seed fibrillation and deposition into diseased tissues.

## Introduction

Intrinsically disordered proteins (IDPs) comprise a class of proteins that defy the structure–function paradigm as they do not have well-defined 3D structures. Rather, they occupy a structural ensemble of rapidly inter-converting conformations, allowing IDPs to carry out important functions during which they often undergo structural transitions^1–5^. It is also recognized that structural plasticity of IDPs is in part responsible for their disproportional prevalence in diseases, including neurodegenerative disorders, cancers, and cardiovascular diseases^6–13^. The misfolding and aggregation of tau, a microtubule-associated IDP, into highly ordered β-sheet-rich fibrils, paired helical filaments (PHFs), that subsequently deposit into neurofibrillary tangles (NFTs) inside neurons are implicated in a range of neurodegenerative disorders, collectively termed tauopathies that include Alzheimer’s disease (AD), Pick’s disease, and frontotemporal dementia with parkinsonism-17^11,14^. Despite our increased understanding of tau physiology and pathology^15–17^, a key feature of tau pathology, tau aggregation, remains to be resolved at the structural and mechanistic level.

The aggregation of natively folded proteins has been found to involve at least two critical steps. The first requires the perturbation of the protein’s native structure to form an aggregation-competent intermediate that proceeds through the formation of a structurally expanded transition state^18–20^. The second step involves the assembly of intermediates into aggregates. The energetics of the two steps are respectively governed by the conformational stability of the protein native state and colloidal stability of the protein solution^21,22^. In contrast to natively folded proteins, IDPs have very little conformational stability and the disordered native state is often stable and resistant to aggregation. Native tau is highly soluble, contains many charged and hydrophilic residues, and shows little tendency for aggregation^10,17,23^. Thus, tau aggregation must proceed through a different mechanism and requires a different type of structural perturbation to render tau aggregation-competent.

The aggregation of tau involves the transition from a disordered monomeric state to a fibrillar state with a highly ordered core. In contrast to folded proteins, it is generally believed that this ordered aggregation pathway first involves the formation of a partially folded aggregation-competent intermediate and proceeds through a structurally perturbed transition state. Depending on the aggregation condition, the transition state could be a more structurally compact conformation compared to the natively unfolded state^24^, or a more specific conformation, such as a perturbed state wherein the protective paperclip-like suprastructure of tau is opened^25^. The aggregation-competent intermediates then irreversibly assemble into higher order aggregates. As the native, disordered state of tau is soluble and stable, energetic barrier for forming the transition state for tau fibrillation is high, resulting in slow tau aggregation. Conditions that favor perturbed conformations of tau or induce disordered-to-ordered transitions, can reduce activation free energy and drive aggregation.

In vitro studies have shown that tau aggregation follows a heterogeneous nucleation-elongation pathway where aggregation is accelerated by polyanionic cofactors, including heparin, RNA, and arachidonic acid micelles^23,26–32^. More recently, a few studies have shown that tau undergoes liquid–liquid phase separation (LLPS) and that the phase-separated tau can initiate tau aggregation^33–35^. Anionic lipid membranes have also been shown to efficiently facilitate tau aggregation^23,26,36,37^ and therefore provide a physiologically relevant context for tau aggregation in vivo. Tau has been shown to favorably interact with anionic membranes^38–41^, although details of the structural changes and assembly associated with membrane binding that lead to fibril nucleation remain to be elucidated. We previously studied the interaction between the full-length wildtype tau, hTau40, with zwitterionic, anionic, and cationic lipid monolayers at the air/water interface^42^. Our results showed that although highly soluble and charged, hTau40 is also highly surface active and preferentially interacted with anionic membranes. Moreover, hTau40 associated with anionic lipid membranes is more conformationally compact compared to its disordered state in solution^42^, indicating a membrane-mediated aggregation pathway for tau assembly.

**Figure 1:**
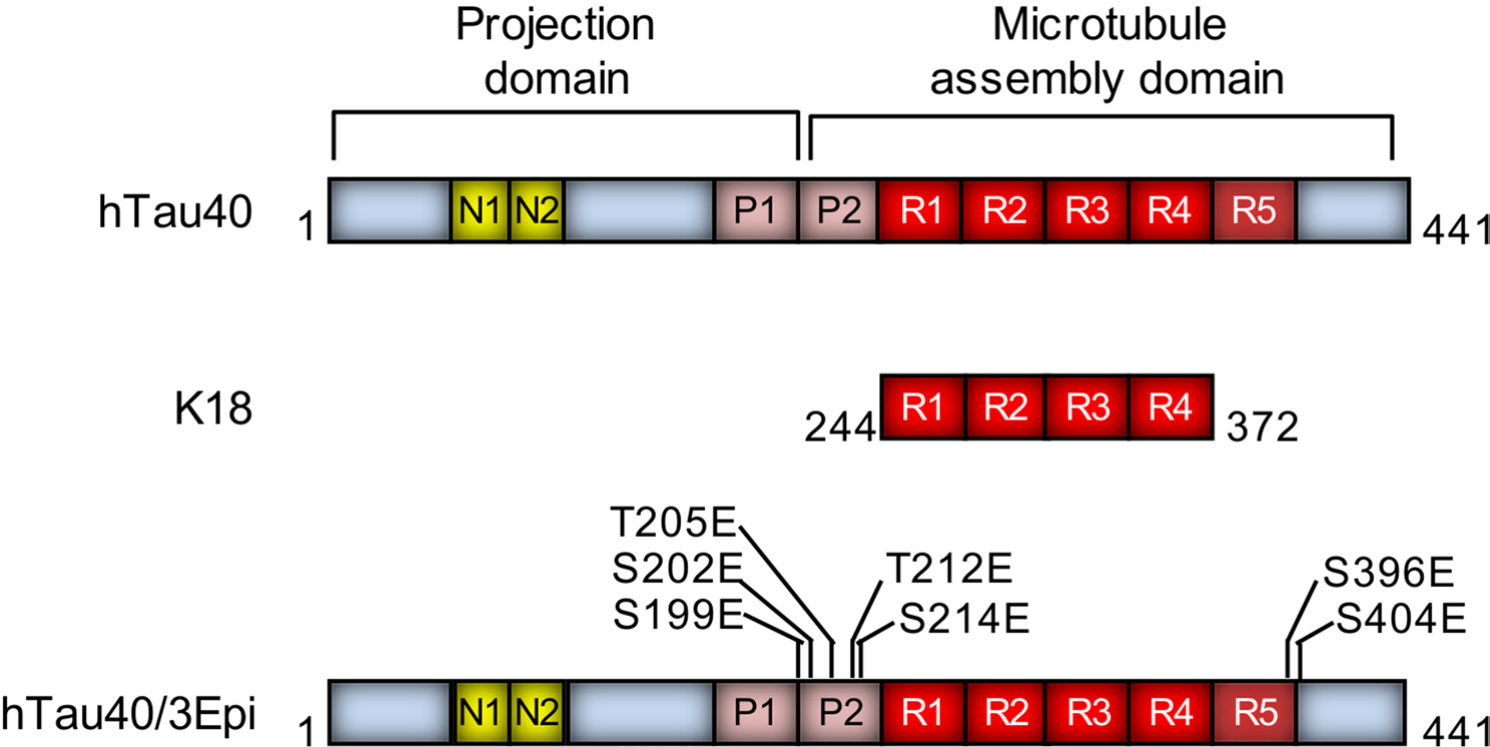
Schematics of domain compositions of the three tau proteins studied. Wildtype hTau40 (441 residues) is the largest human isoform in the central nervous system. Tau construct K18 (Q244-E372) contain the 4 repeats (R1–R4) and hTau40/3Epi is a hyperphosphorylation-mimicking mutant with 7 extra anionic residues in the flanking domains. The serine (S)/threonine (T) to glutamic acid (E) mutations cover key epitopes of 3 antibodies against phospho-tau which require 2 (or 3) adjacent phosphorylation sites. The epitopes are recognized by the antibodies AT8 (residues S199, S202, and T205), AT100 (residues T212 and S214), and PHF1 (residues S396 and S404). These antibodies are used as diagnostic markers for incipient neurodegeneration. Net charges on the tau proteins are: hTau40 =  + 2, K18 =  + 10, and hTau40/3Epi = − 5.

To evaluate the effects of tau domain composition and hyperphosphorylation on tau surface activity and tau-membrane interactions, and to resolve tau structural changes, such as secondary structure formation, that might arise from membrane binding and structural compaction, we investigated the interactions between three different tau proteins (Fig. 1) with anionic lipid membranes. In situ synchrotron X-ray scattering methods were used to resolve high resolution structural details of tau-membrane interactions. The wildtype full length hTau40, the truncated isoform K18, as well as a hyperphosphorylation-mimicking mutant (hTau40/Epi) were used to explore the effects of tau domain composition and hyperphosphorylation on the protein’s surface activity and membrane interactions. K18 (residues Q244-E372) lacks the N-terminal projection domain and the C-terminal tail, but contains the repeat domain (R1–R4) that forms the core of tau fibrils^43^. Phosphorylation of tau is developmentally regulated^44^, and in tauopathies, tau is abnormally hyperphosphorylated, particularly at or near the flanking domains^45^. Phosphorylation in the repeat domain (e.g. in the KxGS motifs) reduces tau affinity to microtubules and triggers detachment from microtubule surface. The interplay between different phosphorylation sites, tau conformation, interactions, and aggregation is still not well understood^46^. In this study, we used a pseudo-hyperphosphorylation mutant of hTau40, hTau40/3Epi, wherein 7 serine and threonine residues in the flanking domains were mutated into anionic glutamic acid residues (Fig. 1). These sites correspond to the epitopes of 3 antibodies (AT8, AT100, and PHF1) that recognize phospho-tau at early stages of neurodegeneration. Although glutamic acid is not a perfect substitute for phosphorylation, it is a reasonable approximation and has the advantage of being controllable compared to incomplete and heterogeneous phosphorylation by various kinases^47^.

Interactions between the three tau proteins with lipid monolayers at the air/water interface were measured. In situ synchrotron grazing incidence X-ray diffraction (GIXD) was used to resolve angstrom-level structural details of tau and membrane at the air/water interface. Our results show that anionic membranes assemble tau into β-sheet-rich oligomers, providing direct evidence of membrane-templated tau misfolding and aggregation.

## Results

### Tau proteins are highly surface and membrane active

We have previously shown that although highly charged and soluble, the full-length hTau40 is highly surface and membrane active^42^. To evaluate if these properties are intrinsic to the protein, we assessed the surface and membrane activities of K18 and hTau40/3Epi proteins. Surface activity was evaluated by measuring surface pressure (π) isotherms of tau adsorption to the air/water interface from a water subphase in a Langmuir trough where π is the magnitude by which the surface tension (γ) of a clean air/water interface (γ_o_) is reduced by the presence of an adsorbate (π = γ_o _− γ) such as proteins or lipids. The air/water interface has been used in both experimental and computational studies as a model hydrophobic/hydrophilic interface to mimic the cell’s organic/aqueous interfaces. In this study, the adsorption of tau to the air/water interface was characterized to assess tau’s propensity to interact with a purely hydrophobic surface, which contributes significantly to its interactions with more complex bio-interfaces, such as membranes.

**Figure 2:**
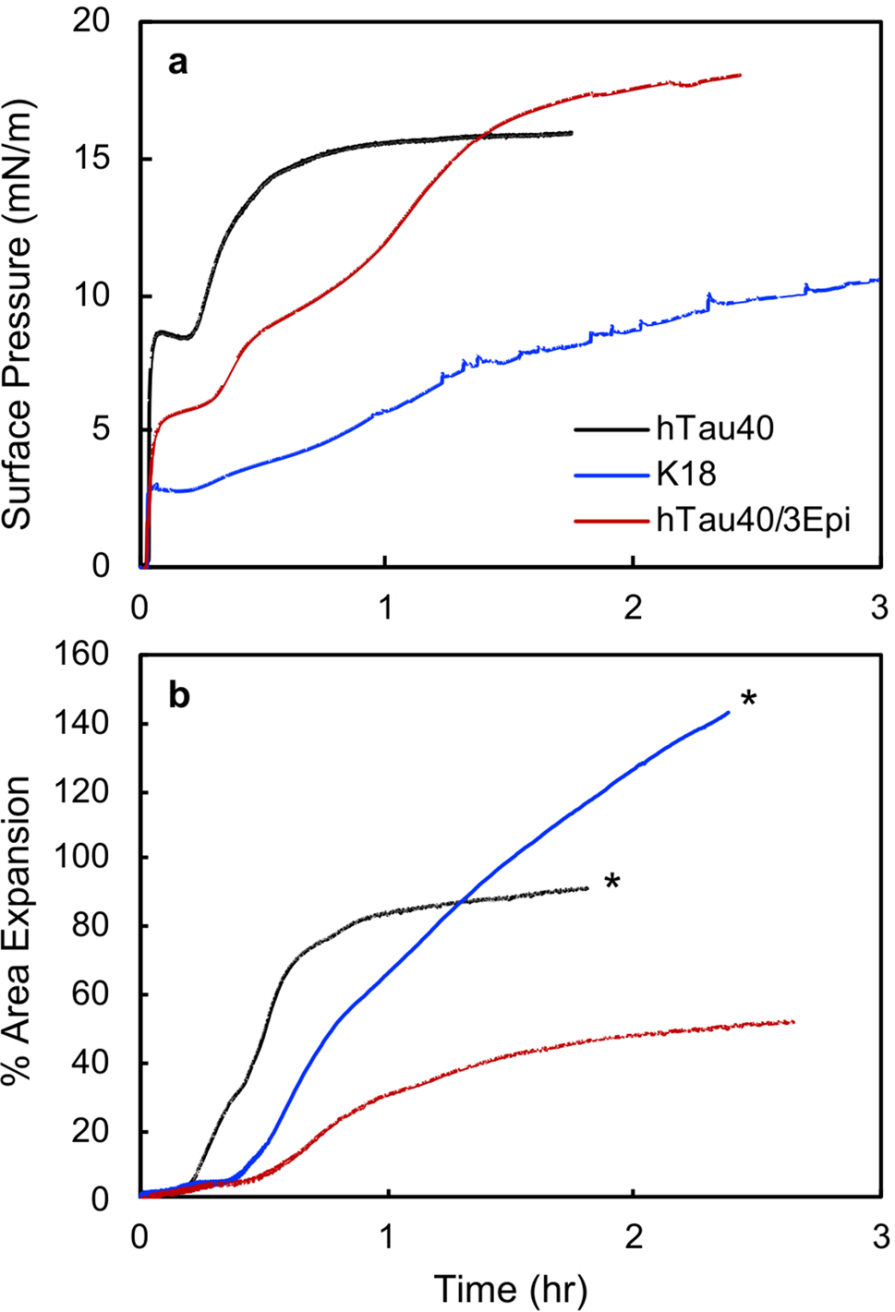
(**a**) Adsorption isotherms of 1 μM tau to the air/water interface at 25 °C. Time 0 corresponds to the time of tau injection into the water subphase of a Langmuir trough. Surface pressure (π) is defined as π = γ_o _− γ where γ_o_ is the surface tension of a clean air/water interface and γ the surface tension of the air/water interface with an adsorbate. (**b**) % area expansion profiles of 1 μM tau inserting into DMPG monolayers held at 25 mN/m on water subphase at 25 °C. A lipid monolayer was first compressed to 25 mN/m, after which tau was injected into the subphase (time = 0). Insertion of tau into the membrane due to favorable tau-lipid interactions results in the expansion of the monolayer surface area. In the two experiments marked by *, the lipid monolayers became completely expanded in the Langmuir trough and further expansions were not measurable.

All tau proteins readily adsorbed to the air/water interface from the bulk causing immediate and rapid increases in π followed by more gradual increases (Fig. 2a). These features reflect different stages of tau adsorption wherein diffusion and adsorption of the protein from the bulk to the interface caused the initial rapid increases in π. Structural rearrangement of the adsorbed tau as well as addition of new tau to the adsorbed layer likely caused further and slower increases in π. Adsorption of the mutant hTau40/3Epi is moderately slower, although the final π reached was slightly higher than that of hTau40. The 7 extra anionic residues of the mutant, which changed tau’s net charge from + 2 to − 5, increased repulsive protein–protein electrostatic interactions and may have contributed to the reduced adsorption rate, but did not alter tau’s accumulation at the interface. The shorter K18 construct also readily adsorbed to the air/water interface, but at an even slower rate and reached a lower π value (Fig. 2a). K18 has a net charge of + 10 and the strong repulsive protein–protein interactions likely caused the slow adsorption rate as well as the lower π value reached. Nonetheless, our results show that the repeat domain of tau, which forms the core of PHFs, is surface active and contributes significantly to the overall surface activity of tau.

Many factors influence protein surface activity. Non-polar residues contribute to hydrophobicity and are expected to strongly favor protein adsorption and influence protein orientation at the air/water interface. Moreover, as multi-point contacts between the protein and the surface are usually required for favorable adsorption^48,49^, conformational flexibility plays an important role in a protein’s surface activity. Structurally flexible, or “soft” proteins readily undergo structural rearrangements at an interface to make more contacts. For natively folded proteins, this type of structural changes usually results in protein denaturation upon surface adsorption. For tau, the disordered native state allows the protein to easily rearrange to maximize desolvation and expose non-polar residues to the air phase. Our results indicate that this driving force is sufficiently strong so that increases in tau-tau repulsion from the 7 extra anionic residues at phosphorylation sites did not reduce tau’s surface activity. In fact, after a slower adsorption rate, hTau40/3Epi reached a higher π compared to hTau40. It is also clear that both the projection domain as well as the repeat domain contribute to tau’s surface activity as the adsorption of the two larger tau proteins resulted in larger π values compared to K18. Overall, our results indicate that tau’s surface activity is an intrinsic property of the “soft” protein, and one that is shared by tau proteins of varying domain or amino acid compositions.

In addition to being surface active, all tau proteins were also membrane active (Fig. 2b). In this study, we utilized lipid monolayers at the air/water interface as our model membrane system to mimic one leaflet of the cell membrane. Although not as physiological as bilayer model membranes, lipid monolayer has the advantage of being able to be precisely controlled in its lipid density and is amenable for high resolution surface characterization methods such as GIXD utilized in this study. Cell membranes contain a large number of different proteins and lipids, some of which are asymmetrically distributed across the two membrane leaflets. The membrane is also compartmentalized into different microdomains that have specific compositions and physio-chemical properties^50^. Major phospholipid components of the neuronal membrane are zwitterionic phosphatidylcholine and phosphatidylethanolamine lipids. Glycolipids, some containing anionic sialic acid groups in the lipid headgroups, and anionic lipids such as phosphatidylserine are also abundant in neuronal membranes^51^. Our previous study showed that hTau40 did not interact with zwitterionic phosphatidylcholine lipid monolayers but favorably interacted with anionic 1,2-dimyristoyl-*sn*-glycero-3-phospho-(1′-rac-glycerol) (DMPG) monolayers^42^. DMPG is not present in neuronal cells, but is used as a mimic for anionic lipids in neuronal cells. In this study, we used pure DMPG lipid monolayers to investigate the interactions and associated structural changes with the tau proteins. Although vastly simplified compared to biological membranes, the pure DMPG membrane can potentially amplify weak protein-membrane interactions so that they are detectable on an experimental time scale.

As shown in Fig. 2b, despite their different charges, each tau protein inserted into an anionic DMPG lipid monolayer held at a constant π of 25 mN/m, causing monolayer area expansions that ranged from 50% to over 140%. This π value was chosen for its relevance to physiological conditions as the lipid packing density of a bilayer membrane has been reported to correspond to that of a monolayer at around 25–30 mN/m^52^. Compared to the cationic hTau40, the anionic hTau40/3Epi inserted into the DMPG monolayer at a slower rate and caused about half the area expansion (Fig. 2b).

Favorable interactions of K18 as well as the related K19 construct with anionic lipid membranes have been attributed to favorable protein-lipid headgroup electrostatic interactions^38,40^. Our results show that despite the overall anionic nature of hTau40/3Epi, the hyperphosphorylation-mimicking mutant readily and favorably interacted with the anionic membrane, demonstrating a strong driving force for tau membrane binding that overcomes electrostatic repulsions. Not surprisingly, the strongly cationic K18 construct caused significantly larger monolayer area expansion compared to hTau40. We note that due to experimental constraints, the insertion of hTau40/3Epi and K18 did not reach final equilibrated states, as the Langmuir trough barriers became fully expanded during these experiments. Thus, further protein insertion, especially for K18, was not measured. Nonetheless, it is clear from our results that although hyperphosphorylation-mimicking mutations reduced tau-membrane interactions, they did not prevent the protein from interacting with and intercalating into the anionic membrane. Truncation of the tau projection domain significantly enhanced the interactions of the repeat domain with the DMPG membrane as K18 was the most membrane active.

### Tau proteins disrupt membrane morphology

**Figure 3:**
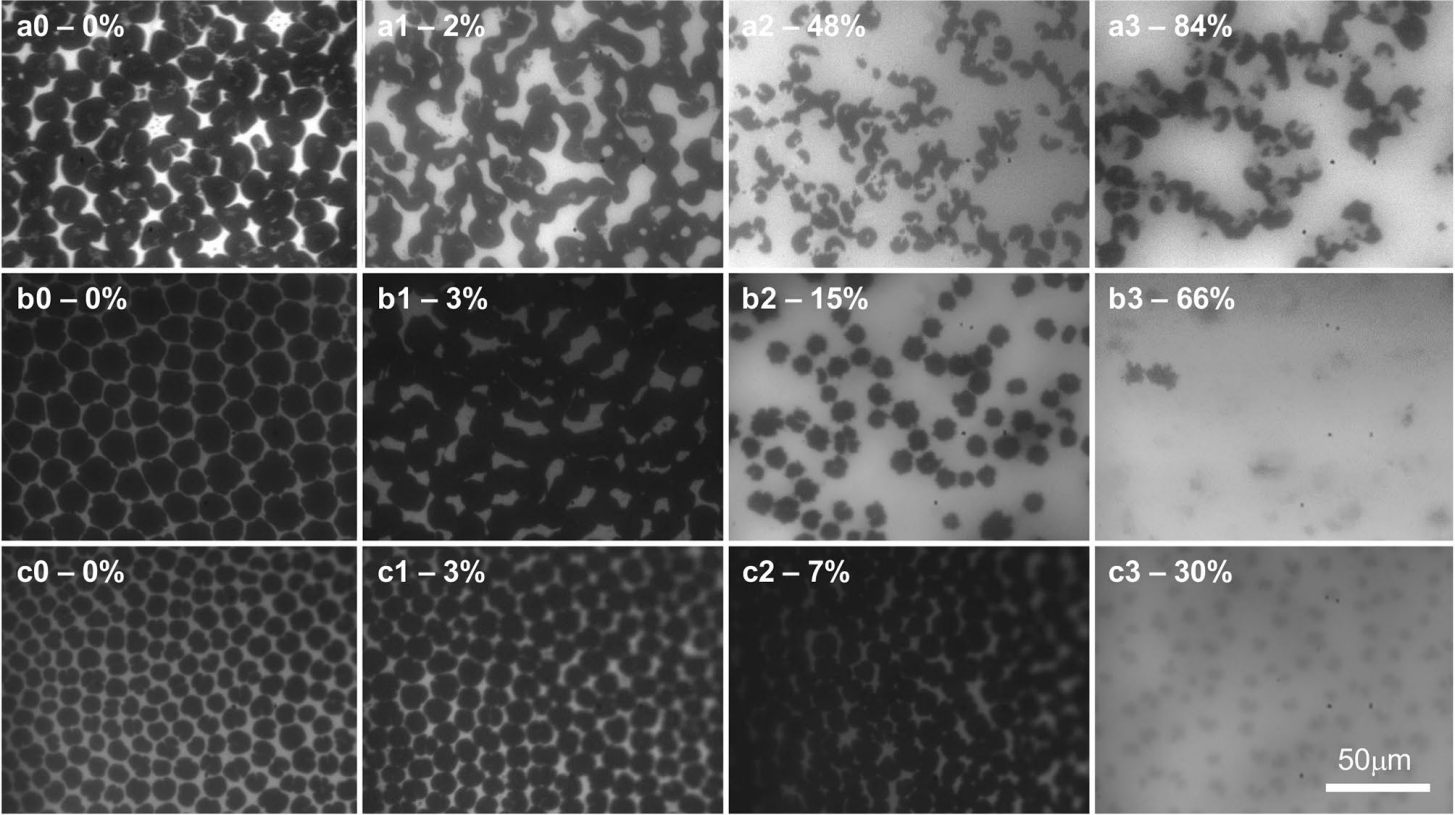
Fluorescence microscopy (FM) images of DMPG lipid monolayers at 25 mN/m before (**0**) and 10 (**1**), 30 (**2**), and 60 (**3**) minutes after injecting hTau40 (**a**)^42^, K18 (**b**) or hTau40/3Epi (**c**) into the subphase. FM images of hTau40 (a0–a3) were reproduced from Jones et al.^42^. % trough area expansion due to tau membrane insertion at the time points are also indicated. Head group-labeled fluorescent dye TR-DHPE (0.5 mol%) was included in the lipid monolayer to provide fluorescence contrast. Dark regions of the monolayer correspond to dye-excluded liquid-condensed (LC) domains whereas light, or fluorescent region of the monolayer corresponds to disordered liquid-expanded (LE) phase of the monolayer where the TR-DHPE dye is mixed with the DMPG lipids.

To assess changes in lipid monolayer morphology caused by the insertion of the tau proteins, fluorescence microscope (FM) images of the lipid monolayer were taken before and at various time points after the injection of tau during constant-π insertion assays. Representative images are shown in Fig. 3 and percent area expansion is indicated for each image. At 25 °C, the DMPG monolayer on water subphase undergoes a liquid-expanded (LE) to liquid-condensed (LC) phase transition at around 17 mN/m^53^. Because the bulky lipid dye molecules, Texas Red 1,2-Dihexadecanoyl-*sn*-Glycero-3-Phosphoethanolamine (TR-DHPE) (0.5 mol%), are preferentially excluded from the LC phase, it appears as dark domains whereas the fluid LE phase containing the dye is bright.

As shown, DMPG monolayer at 25 mN/m contained predominantly dark LC phase domains (Fig. 3a0, b0, c0). At 10 min after tau injection where minimal protein insertion had occurred (Fig. 3a1, b1, c1), the LC domains changed from a well-defined and circular shape to less defined shapes where many of the LC domains had fused. These changes indicate decreased line tension around the LC domains due to the association of tau to the lipid monolayer but before any significant tau insertion had occurred. At 30 min after injection, where significant insertion had occurred for hTau40 and K18, 48% and 15% trough area expansions, respectively, the morphology of the monolayer changed drastically (Fig. 3a2, b2). The ratio of dark to light regions was reduced and the LC domains became smaller, especially for hTau40. These changes indicate that tau insertion into the disordered phase had pushed the LC domains apart, as well as disrupted the LC phase. hTau40/3Epi inserted into the membrane at a slower rate and caused about 7% area expansion 30 min after injection. Although the monolayer contained primarily LC domains, the domains fused and became less defined (Fig. 3c2), similar to those observed for hTau40 and K18 insertion at the 10 min time point with low insertion levels (Fig. 3a1, b1). At 1-h post injection, the morphology of the lipid monolayer incubated with hTau40 (Fig. 3a3) did not significantly differ from that at 30 min post injection, although area expansion almost doubled during that time period. However, morphologies of monolayers from K18 and hTau40/3Epi insertion significantly changed. The LC domains became almost completely disrupted where distinct domains boundaries were no longer present.

The FM images of the lipid monolayer indicate that the association of the tau proteins to the lipid monolayer caused observable changes to the monolayer morphology even in the absence of significant insertion at earlier time points. The LC phase domains were perturbed with hTau40 insertion, but the domains retained their distinct boundaries. However, insertion of the more highly charged proteins K18 and hTau40/3Epi, caused almost complete destruction of the LC phase, indicating that these two proteins were more membrane disruptive than hTau40.

### Anionic membrane templates misfolding and assembly of tau into β-sheet-rich aggregates

Using synchrotron GIXD, we monitored the time-dependent in situ insertion and accumulation of the tau proteins with DMPG monolayers. A lipid monolayer at 25 mN/m was first formed and an aliquot of tau was then injected into the water subphase underneath the monolayer. In these experiments, Langmuir trough surface area was held constant. As such, insertion of tau into the monolayer caused increases in π; final π values ranging from 34 to 46 mN/m were obtained for the different tau proteins. GIXD measurements were collected before (*t* = 0) and at approximately 2.5 and 12 h after tau injection. As shown previously^42^, at 25 °C DMPG monolayer contained ordered LC phase giving rise to in-plane *Bragg peaks* which allowed us to track changes to lipid packing during tau insertion.

In GIXD experiments, the X-ray beam strikes the surface at an incident angle below the critical scattering angle for total external reflection. At this angle, an X-ray evanescent wave is generated and penetrates a few nanometers into the bulk liquid^54^. The wave travels along the surface and Bragg scatters from two dimensional (2D) ordered molecular arrangements at the air/water interface. GIXD measurements therefore provide in-plane (i.e. in the plane of the monolayer) structural information on the diffracting, or ordered, portion of the film. In our experiments, the lipid alkyl tails in the LC phase and ordered tau assemblies give rise to diffraction peaks. The reciprocal space GIXD patterns from the 2D ordered structures (*Bragg peaks* and *rods*) can be analyzed to provide complete structural information about molecular arrangement of lipids and the interacting proteins^55–58^.

**Figure 4:**
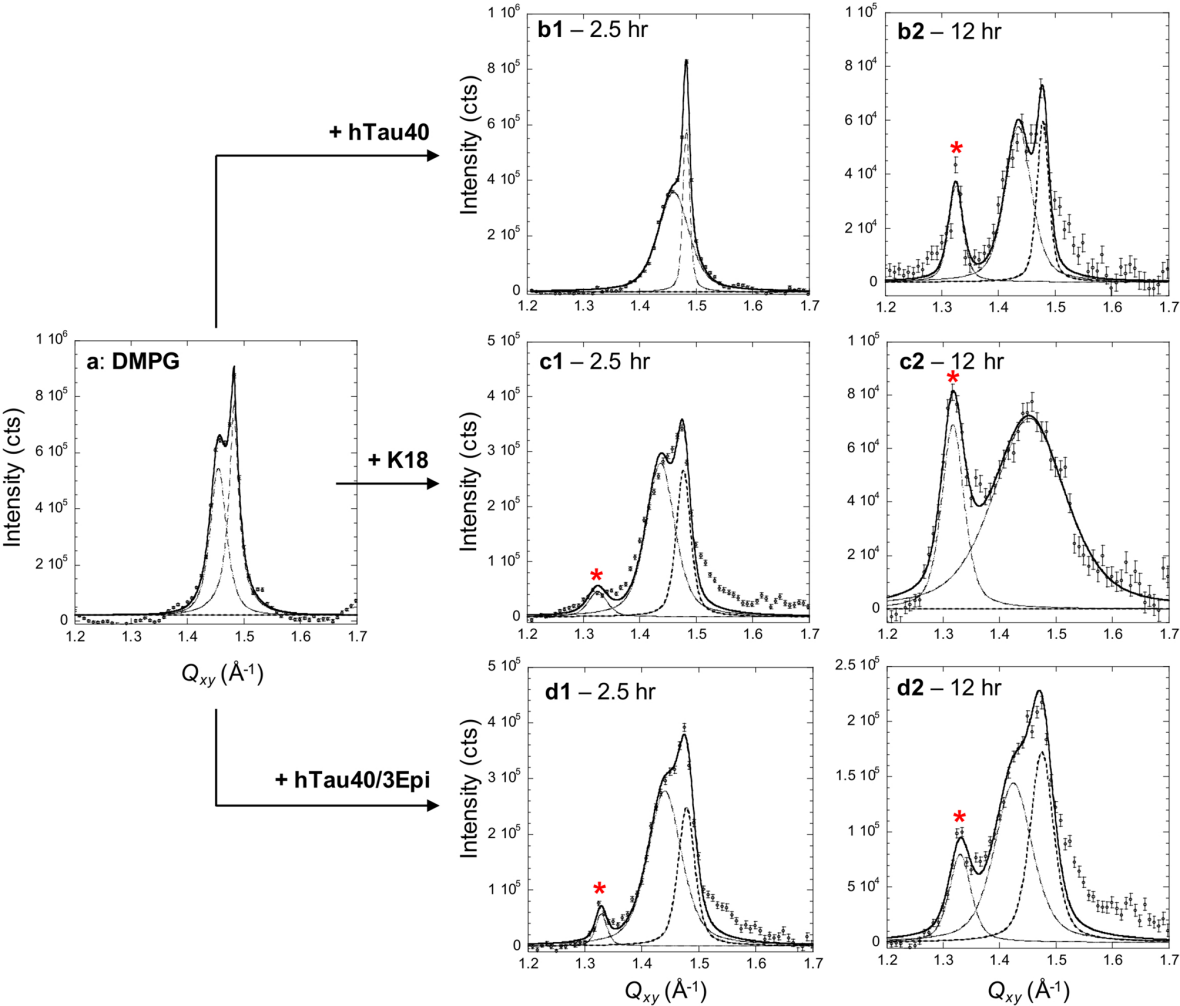
*Bragg peaks* from DMPG monolayer at the air/water interface at 25 mN/m and 25 °C before (**a**) and *t*_1_ = 2.5 and *t*_2_ = 12 h after the injection of hTau40 (**b**), K18 (**c**) and hTau40/3Epi (**d**) proteins. The *Bragg peaks* were fitted using the sum of two Voigt profiles (solid line) and de-convoluted into separate peaks (dashed lines) corresponding to {1,0} + {0,1} and {1, − 1} *Bragg peaks* in the distorted hexagonal 2D unit cell. *Bragg peaks* were obtained by integrating over the − 0.05 Å^−1^ ≤ *Q*_*z*_ ≤ 0.75 Å^−1^ region. Red asterisks indicate *Bragg peaks* associated with ordered β-sheet structures of the tau protein. Structural parameters extracted from GIXD and *Bragg rods* are presented in Supplemental Information.

Figure 4 shows GIXD data obtained for DMPG monolayer before (Fig. 4a) and after injecting each tau in the subphase (Fig. 4b–d). Structural parameters extracted from the diffraction peaks are summarized in Tables S1, S2, and S3 in Supplemental Information. Figure 4a shows two *Bragg peaks* resulting from the LC phase of the DMPG monolayer, indicating a distorted hexagonal 2D arrangement of the alkyl tails^58^. The broader peak at lower *Q*_xy_ is the superposition of two {1,0} + {0,1} Bragg reflections and the sharper peak at higher *Q*_xy_ is the {1,− 1} reflection. Peak broadness is inversely proportional to the average size of the 2D ordered domains, or coherence length *L*_xy_^59^, which are 170 and 310 Å for the {1,0} + {0,1} and {1,− 1} directions, respectively (Table S1B). Insertion of hTau40 caused significant changes to the lipid packing (Fig. 4b1, b2). The intensities of the lipid diffraction peaks decreased where *L*_xy_ values were 100 and 240 Å for the {1,0} + {0,1} and {1,− 1}directions, respectively, and the integrated intensity reduced by 87% after 12 h of tau incubation (Figs. 4b2, 5b, Table S1). Strikingly, decrease in lipid packing was accompanied by the appearance of a new diffraction peak marked with a red asterisk at *Q*_xy_ = 1.34 Å^−1^. This *Q*_xy_ value corresponds to a *d*-spacing of 4.74 ± 0.02 Å, which exactly matches the average distance between β-sheet arrangements in tau fibrils^60^. *L*_xy_ of the β-sheet ordering was around 200 Å (Table S1C), indicating that there were approximately 42 tau β-strands arranged in positional registry. Our results thus show that the insertion of hTau40 into the DMPG monolayer induced two concomitant structural changes, tau-induced disruption of lipid-packing and membrane induced assembly of the disordered tau protein into ordered β-sheet aggregates.

**Figure 5:**
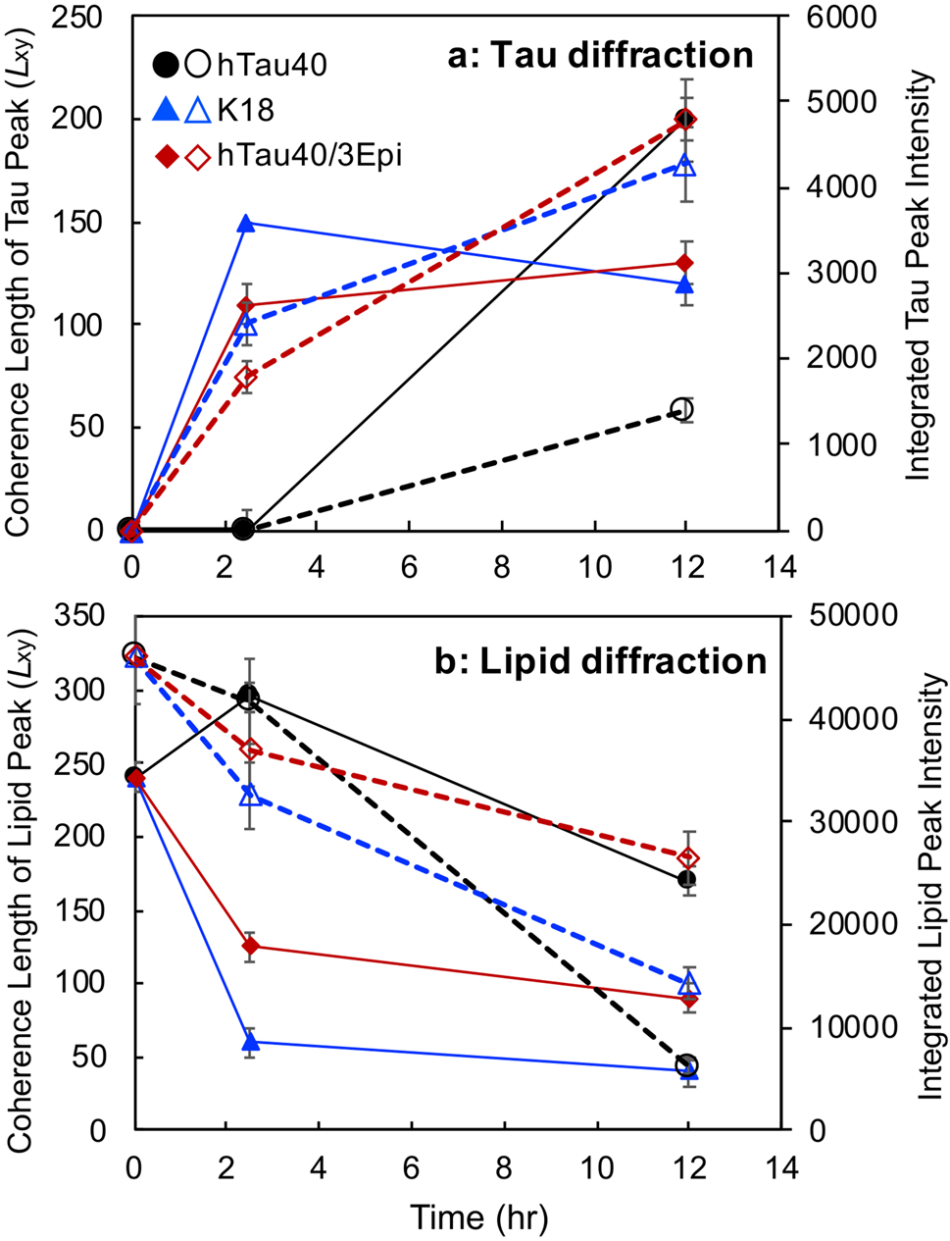
Coherence length (*L*_xy_ in Å) (filled symbols and solid lines) and integrated intensities (unfilled symbols and dashed lines) of the tau *Bragg peaks* at *Q*_xy_ = 1.34 Å^−1^ (**a**) and lipid *Bragg peaks* (**b**) extracted from GIXD measurements of tau insertion into DMPG monolayers. For lipids, averaged *L*_xy_ values of the {1,0} + {0,1} and {1,− 1} *Bragg peaks* were plotted.

GIXD results for K18 and hTau40/3Epi interacting with DMPG monolayers also showed the two salient features of hTau40-membrane binding, membrane-templated β-sheet-rich tau aggregate formation and tau-induced membrane disruption (Figs. 4c, d, 5). However, important differences were also observed. K18 caused rapid decreases in the intensities of the lipid diffraction peaks where the two original peaks converged into one broad reflection 12 h after K18 injection (Fig. 4c1, c2), which had a *L*_xy_ value of only 40 Å. The integrated intensities of the lipid peaks reduced to 70% and 30% of the original value at 2.5 and 12 h, respectively, after K18 injection. These results indicate that the LC domains became more disrupted with K18 insertion compared to hTau40. This finding is consistent with FM images where K18 caused the most disruption to LC domains at the morphological scale. The remaining ordered structures corresponded to short-ranged hexagonal packing of lipid tails with increased unit cell vectors from 4.93 to 5.00 Å, indicating an increase in area per lipid molecule in the monolayer, even as the π of the film was significantly higher, at 46 mN/m. Importantly, the β-sheet ordered tau aggregates appeared earlier for K18 than hTau40, where the *Bragg peak* at *Q*_xy_ = 1.34 Å^−1^ was already visible at 2.5 h. After 12 h of incubation, *L*_xy_ value of the ordered K18 peak did not significantly change but the intensity of this peak increased two-fold, indicating that while the size of the ordered K18 aggregates did not grow, their number doubled from 2.5 to 12 h of incubation (Fig. 4c1, c2, 5, Table S2).

Similar to K18, hTau40/3Epi caused significant reductions to the amount of ordered LC phase in the DMPG monolayer and gave rise to an in-plane β-sheet diffraction peak (marked by asterisks) (Fig. 4d1, d2). Despite the strong repulsive protein–protein and protein–lipid electrostatic interactions, the mutant formed the tau diffraction peak earlier compared to hTau40. By 2.5 h of incubation (Fig. 4d1), the tau diffraction peak was visible and the amount of tau aggregates grew by almost three-fold by 12 h (Figs. 4d, 5, Table S3).

In addition to analyzing the *Bragg peaks*, the analysis of their *Bragg rods* was also carried out to obtain the lengths of the coherently scattering moieties participating in the Bragg reflection (*L*_c_). For lipid scattering, *L*_c_ is the length of the coherently scattering portions of the alkyl tails measured along their backbones. For protein scattering, *L*_c_ is the length of the coherently scattering β-sheet portion of the protein layer. Although the *Bragg rod* data were in general noisy (Figs. S1–S3 in Supplemental Information), we were able to extract *L*_c_ values for the protein scattering peaks and they ranged from 4.5 to 7.2 Å. The height of the β-sheet strand has been reported to be approximately 10 Å^59^. Our analysis thus indicates that the β-sheet moiety that coherently participate in the Bragg reflection is one layer of β-sheets.

In contrast, ordered aggregates were not observed for tau films adsorbed at the air/water interface as no in-plane diffraction peak was observed (Fig. S4). Concentrating tau at the air/water interface is thus insufficient to induce β-sheet oligomer formation. Favorable interactions between tau and anionic lipids are thus necessary to template tau misfolding and assembly into β-sheet-rich oligomers.

## Discussion

**Figure 6:**
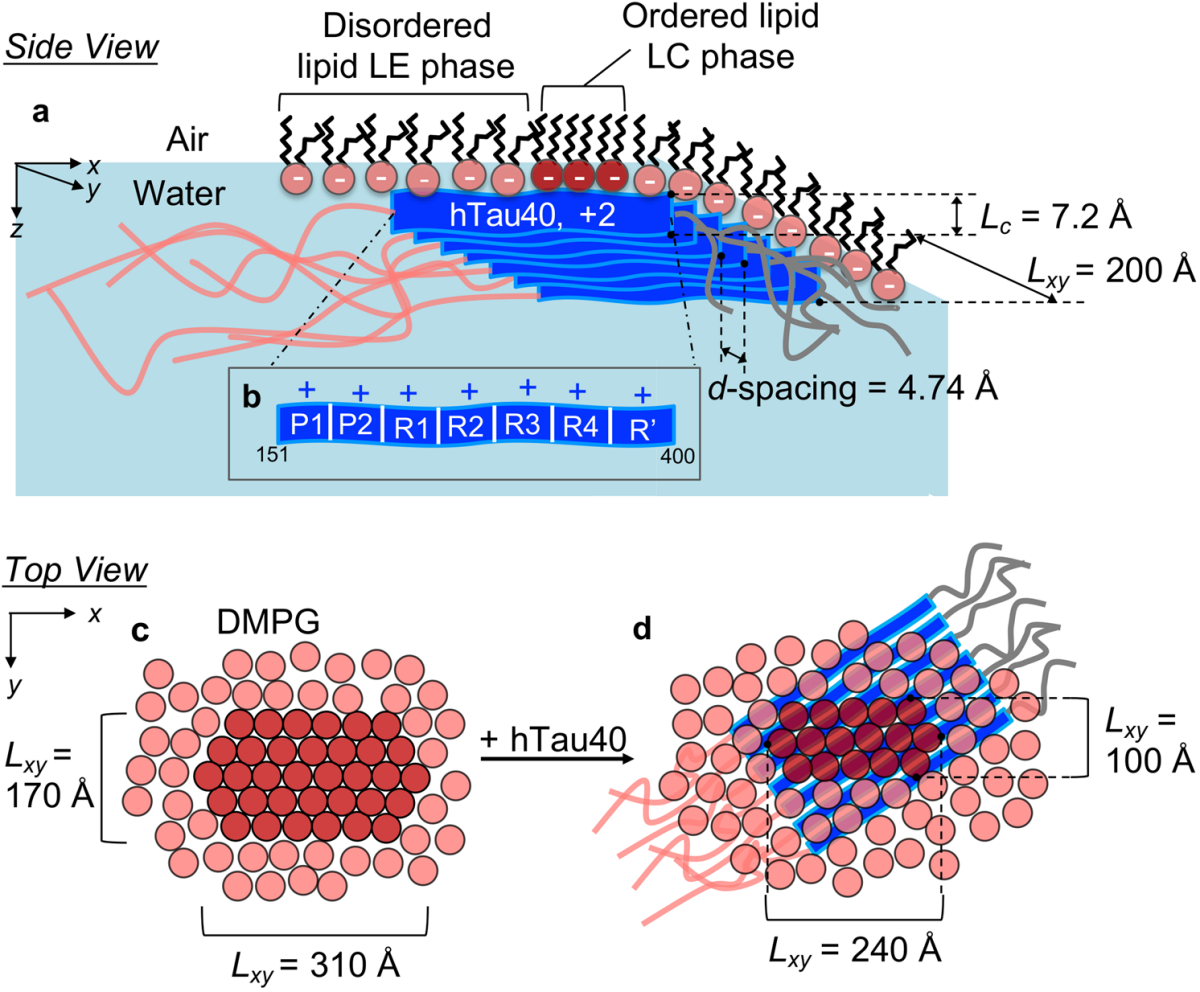
Schematics of a β-sheet enriched hTau40 oligomer templated by the anionic DMPG monolayer. (**a**) and (**b**) side views (in the plane of the membrane) shows an ordered tau oligomer associated with the anionic DMPG monolayer at the air/water interface (**a**). *d*-spacing and coherence lengths (*L*_*xy*_) shown are for the hTau40 oligomer. *L*_*c*_ is the out-of-plane length of the coherently scattering moiety of the hTau40 assembly. A zoomed in schematic illustrates the cationic tau domains P1 and P2) (adopted from Wang et al.^15^) that might favorably interact with the anionic lipids (**b**). (**c**, **d**) top views (perpendicular to the membrane) of the membrane before (**c**) and after (**d**) hTau40 association. The *L*_*xy*_ values in these schematics are those of the ordered lipid domains for the {1,0} + {0,1} and {1,− 1} Bragg peaks. The orientation of the tau β-sheet arrangements vis-à-vis the orientation of the DMPG lattice cannot be determined from the experimental data. Schematics for K18 and hTau40/3Epi can be found in Figures S5 and S6 in Supplemental Information, respectively.

Taken together, our results yielded molecular level insights into the structure and dynamics of tau association with anionic membranes (Fig. 6). As illustrated in Fig. 6a, hTau40 binding and insertion into the lipid membrane resulted in the structural compaction^42^ and misfolding of the disordered tau into aggregates wherein the protein is arranged in an extended β-sheet conformation that is one layer thick. Our previous study showed a complete disruption of DMPG lipid packing upon hTau40 insertion and did not observe a protein diffraction peak^42^. The differences between the findings of the two studies may be due to differences in the experimental conditions. Instead of constant π insertion at 25 mN/m, X-ray scattering experiments conducted in this study were carried out under constant-area conditions where π greatly increased during tau insertion. Higher surface pressures forced the lipids to be in the LC phase, and perhaps also reduced the extent by which associated tau disrupted lipid packing. To observe tau structural features that might arise with the incubation with lipid membranes, the current study was conducted at longer incubation times, up to 12.5 h post tau injection, compared to the ~ 5 h incubation time used in the previous study. As shown, no diffraction peak was observed for hTau40 incubated with DMPG monolayer after 2.5 h of incubation. But after 12 h of incubation at higher surface pressures, a clear tau β-sheet diffraction peak was observed.

K18 and the hyperphosphorylation-mimicking mutant similarly formed β-sheet-rich aggregates at the membrane surface, but compared to hTau40, they formed earlier and larger numbers of aggregates were formed (Fig. S5 and S6). Concomitant to tau binding, lipid packing in the membrane became disrupted where the size of LC phase domains significantly decreased (Fig. 6c, d). Consistent with membrane morphology visualized by FM, K18 and hTau40/3Epi disrupted lipid packing in the membrane to greater extents (Figs. S5b, S5c, S6b, and S6c) compared to hTau40, implicating possibly higher levels of toxicity of these two tau proteins.

The remarkable observation that all tau proteins tested formed β-sheet rich aggregates at the anionic membrane surface confirms results from earlier studies that showed that the nucleation of tau fibrils is strongly enhanced by lipid membrane^23,26,36,37^. For the positively charged hTau40 and K18, our results show that the anionic membrane enhances protein–protein interactions by charge screening and concentrating the protein at the membrane surface^38,40^. Although the structural methods utilized in this study do not give domain or residue-specific information about tau-lipid binding, we suspect that the cationic repeat and flanking domains (P1 through R’) of hTau40 interact with the anionic lipid headgroups (Fig. 6b) while the anionic N-terminus and the neutral C-terminus do not interact with the membrane. This domain interaction model is further supported by the finding that the repeat domain alone (K18) strongly interacts with and forms β-sheet-rich aggregates at the anionic membrane surface. The repeat domain plays major roles in both tau’s physiological function and aggregation and forms the core of PHFs. In particular, two lysine-containing hexapeptide segments, ^275^VQIINK^280^ (PHF6*) and ^306^VQIVYK^311^ (PHF6) located in the second and third repeat units of the repeat domain, respectively, have been identified as aggregation promoting β-sheet forming motifs^61–63^. Our finding that the β-sheet-rich aggregates are only one sheet thick indicates that the backbone of the β-sheet strands is positioned along the membrane surface such that alternate side chains of residues in the β-strand point upwards toward the membrane. Such an arrangement might promote favorable electrostatic interactions between cationic lysine (K) side chains around the PHF6* sequence and anionic lipid headgroups. There are two lysine residues in the PHF6* sequence, K274 and K280, and they are about 20 Å apart, based on a residue-residue distance along a β-strand of 3.5 Å. Given that the tail–tail distance of the lipids in the membrane is about 5 Å, approximately three lipids span the cationic lysine-to-lysine β-strand backbone distance.

The recent tau LLPS studies show that tau demixing into protein-rich droplets is facilitated by crowding agents^33–35^ and that the additional presence of polyanions such as heparin and RNA promotes tau fibrillation in the droplets. As LLPS appears to be a fundamental mechanism for organizing intracellular space^64^, the LLPS of tau can lead to subcellular regions of high local tau concentration, which combined with other aberrant events such as hyperphosphorylation may lead to tau aggregation^33^. It also appears that LLPS alone is not sufficient and also not a necessary condition for tau aggregation. Results from this study demonstrate that the anionic lipid membrane can potentially serve both roles, concentrating tau at the membrane surface as well as providing specific lipid-protein interactions that template tau aggregation. In fact, it appears that tau incubated with an anionic lipid membrane over time forms a macroscopic gelatinous material at the air/water interface (Video S1 in Supplemental Material). This viscous surface gel formation was not observed for lipid membranes alone at the air/water interface, nor for an anionic lipid monolayer incubated with the amyloid-β (Aβ) peptide that also inserted into the membrane and formed a β-sheet ordered film at the membrane surface^65,66^ (Video S2 in Supplemental Information). Although details of the gel formation are not yet clear, our observations suggest that tau phase separation may have occurred and can be induced by anionic membranes at a tau concentration as low as 1 μM.

Our finding that the anionic DMPG membrane also templated β-sheet formation of the anionic hTau40/3Epi mutant points to a complex role of the membrane in mediating protein–protein interactions and misfolding. In addition to electrostatic interactions and concentration effects (e.g., higher local protein concentrations at membranes or in phase-separated tau droplets^36^), specific lipid interactions and/or interactions with the hydrophobic lipid tails could also play an important role at facilitating tau-tau interactions and β-sheet assembly.

In conclusion, despite being highly charged and soluble, the flexible nature of the tau proteins also causes them to be highly surface active and all tau proteins favorably interacted with lipid monolayers composed of anionic DMPG lipids at the air/water interface. The K18 repeat domain construct exhibited the strongest interaction with the DMPG membrane and a hyperphosphorylation-mimicking mutant of tau, which has relevance to disease states, retains this affinity to anionic lipid membrane even though it has a high net negative charge. Strikingly, binding to DMPG monolayers gave rise to in-plane protein diffraction peaks which corresponded to one layer of about 40 β-strands in positional registry. Thus, the membrane templated the formation of β-sheet rich tau oligomers at the membrane surface. Compared to the wildtype hTau40, K18 and phospho-mutant hTau40/3Epi formed β-sheet oligomers earlier and at larger numbers.

As tau aggregation is believed to be driven by a transition from random coil to β-sheet structure, our study shows that the lipid membrane effectively catalyzed this structural transition for further growth into mature fibrils. In-plane protein diffraction peaks were not observed for tau adsorbed at the air/water interface. Thus, the accumulation of the protein, or increasing protein local concentration, alone is insufficient to induce tau β-sheet formation. This is in contrast with our previous findings that the Aβ40 peptide formed in-plane diffraction peaks both adsorbed at the air/water interface^67^ and inserted into anionic lipid membranes^65,66^. Although the exact nature of tau-membrane interactions that leads to tau structural re-organization and assembly into extended β-sheet structures remains to be resolved, the effect is membrane specific, but not tau domain composition-specific. Our findings support a general tau aggregation mechanism wherein tau’s inherent surface activity drives tau–lipid membrane interactions, inducing the misfolding and self-assembly of tau into β-sheet enriched oligomers that could subsequently seed tau fibril growth and deposition into diseased tissues. Concomitant with misfolding of the tau protein, tau-membrane interactions also result in the disruption of lipid packing in the membrane, pointing to a possible membrane disruption-based toxicity pathway of tau oligomers.

## Methods

### Materials

hTau40, K18 and hTau40/3Epi were expressed and purified as previously described^47,68,69^. 1,2-dimyristoyl-sn-glycero-3-[phospho-rac-(1-glycerol)] (DMPG) was purchased from Avanti Polar Lipids (Alabaster, AL). Lipid stock solutions ranged from 2 to 10 mg/ml were prepared by dissolving lipids in chloroform (Fisher Scientific, Hampton, NH) containing 10 vol% methanol and then diluted to 0.2 or 0.5 mg/ml for spreading solutions. For fluorescence microscopy, the head group-labeled fluorescent dye Texas Red 1,2-dihexadecanoyl 3-phosphoethanolamine (TR-DHPE) (Molecular Probes, Eugene, OR) was first dissolved in chloroform and subsequently added to lipid spreading solutions at 0.5 mol%. All lipid solutions were stored at − 20 °C in glass vials. All water used was purified with a Milli-Q Ultrapure water purification system (Millipore, Bedford, MA).

### Surface activity and membrane insertion measurements

To evaluate the surface activity of the tau proteins, π of the air/water interface was measured. The experiments were carried out at 25 °C using a MiniMicro Langmuir trough (KSV Instruments Ltd., Finland) with 45 mL of water as the subphase. A Wilhelmy plate sensor measured π of the air/subphase interface. π is defined as = γ_o _– γ, where γ_o_ is the surface tension of a clean air/water interface and γ is the surface tension of the air/water interface with adsorbed protein. The trough had a working surface area of 86.39 cm^2^. Before injecting protein into the subphase, barriers were partially closed to give a total surface area of 45 cm^2^. A gastight glass microsyringe (Hamilton, Reno, NV) was used to inject 1 mL of 45 μM tau to achieve a tau concentration of 1 μM in the subphase.

To evaluate the interaction between tau and membranes, the insertion of tau into lipid monolayers at the air/water interface held at 25 mN/m was measured in a Langmuir trough as previously described^70^. All experiments were carried out on water subphase, at 25 °C, and at 1 μM tau concentration in the subphase. An aliquot of tau was injected into the subphase underneath the monolayer and allowed to equilibrate with the lipid monolayer. Favorable tau–lipid interactions leading to the insertion of tau into the lipid monolayer caused an expansion of the monolayer surface area. The percent area expansion, or the change in the effective area per lipid molecule, is defined as % ΔA/A = 100(A − A_i_)/A_i_, where A is the surface area at time t and A_i_ is the initial surface area of the monolayer prior to the injection of the protein at 25 mN/m. Videos of the protein-lipid film at the air/water interface was taken with a Nexus 5X model LG-H790 smartphone.

### Fluorescence microscopy

To visualize lipid monolayer morphological change during tau insertion, the Langmuir trough was positioned on top of a motorized stage of an inverted fluorescence microscope (Olympus IX 71) with a 50 × objective centered on a quartz window in the bottom of the trough. A 100 W mercury lamp was used for fluorescence excitation. Fluorescence images were collected by a QImaging camera (EXi Blue, QImaging Photometrics) and analyzed using the software QCapture Pro. TR-DHPE (0.5 mol%) was included in the spreading solution.

### GIXD measurements

GIXD measurements were collected at the BW1 beamline (HASYLAB, DESY, Hamburg, Germany) for tau insertion into DMPG monolayers. The synchrotron X-ray beam was monochromated to a wavelength (λ) of 1.30 Å by Bragg reflection from a Beryllium (200) monochromator crystal in Laue geometry. All experiments were carried out in an ultra-small volume Langmuir trough liquid diffractometer (20 mL subphase volume) at 25 °C and 1 μM tau concentration in water. The trough was temperature controlled and equipped with a Wilhelmy balance for surface pressure measurements. Unlike the constant surface pressure tau insertion experiments carried out in the previous section, trough experiments for GIXD measurements were carried out under constant surface area condition. Lipids were spread at the air/water interface to achieve an equilibrated surface pressure of 25 mN/m. An aliquot of tau was then injected into the trough. Insertion of tau into the lipid monolayer caused increases in π.

The X-ray beam strikes the surface at an incident angle of 0.11°, which corresponds to a *Q*_z_ = 0.85*Q*_c_, where *Q*_c_ = 0.02176 Å^−1^ is the critical scattering vector for total external reflection. At this angle, the incident wave is totally reflected, and the refracted wave becomes evanescent with penetration depth of approximately 100 Å which maximizes surface sensitivity. The X-ray beam footprint on the liquid surface was approximately 2 × 50 mm^2^, bigger than the width of the ultra-small Langmuir trough used. This caused over illumination of the sample and small increases in the scattering background. The scattered intensity was measured by scanning over a range of horizontal scattering vectors, *Q*_xy_^55,71^. Bragg peaks are intensity resolved in the *Q*_xy_-direction and integrated over channels along the z-direction (normal to the liquid subphase) in the 1-D position sensitive detector. The position of the maxima of the Bragg peaks, *Q*_xy_^max^, were used to calculate the repeat distances *d* = 2π/*Q*_xy_ of the 2-D lattice. The widths of the peaks, corrected for the instrument resolution, were used to determine the 2-D in-plane coherence length, *L*_xy_. The intensities resolved in the *Q*_*z*_ direction, but integrated over the *Q*_*xy*_ range of the Bragg peaks, so called Bragg rods, were also recorded and analyzed. The analysis of the Bragg rods provides the length (*L*_c_) of the coherently scattering moieties participating in the Bragg reflection. For lipid scattering, *L*_c_ is the length of the coherently scattering portions of the alkyl tails measured along their backbones. For protein β-sheet scattering, *L*_c_ is the length of the molecular moiety participating in coherent scattering.

## Supplementary information

Supplementary Information.

Supplementary Video 1.

Supplementary Video 2.

## Data Availability

The data that support the findings are available from the corresponding author upon reasonable request.
