# Supplementary material for: Lipid membrane templated misfolding and self-assembly of intrinsically disordered tau protein
Source: Sci Rep. 2020 Aug 7;10:13324. doi: 10.1038/s41598-020-70208-6 (PMC7414892; doi:10.1038/s41598-020-70208-6)
Supplement: Supplementary file 1 — Supplementary Information. [file 41598_2020_70208_MOESM1_ESM.docx]

**Supplementary Information**

*for*

**Lipid membrane templated misfolding and self-assembly of intrinsically disordered tau protein**

Jaroslaw Majewski*‡^+^, Emmalee M. Jones†‡, Crystal M. Vander Zanden‡§, Jacek Biernat¶#, Eckhard Mandelkow¶#, Eva Y. Chi‡

*Division of Molecular and Cellular Biology, National Science Foundation, Alexandria, VA 22314, USA

†Nanoscience and Microsystems Engineering Graduate Program, University of New Mexico, Albuquerque, New Mexico, 87131, USA

‡Department of Chemical and Biological Engineering and Center for Biomedical Engineering, University of New Mexico, Albuquerque, New Mexico, 87131, USA

§Department of Chemistry and Biochemistry, University of Colorado at Colorado Springs, Colorado Springs, CO 80918

¶Center for Neurodegenerative Diseases (DZNE), Bonn 53127, Germany

#CAESAR Research Center, Bonn 53175, Germany

^+^ Theoretical Biology and Biophysics Division, Los Alamos National Laboratory, Los Alamos, New Mexico 87545, United States

**Figure S1**: *Bragg peaks* (top row) and *rods* (bottom row) of a DMPG monolayer at the air/water interface at 25 mN/m and 25°C before (A, A’) and *t*_1_ = 2.5 hr (B, B’) and *t*_2_ = 12 hr (C, C’ and C”) after the injection of hTau40. *Bragg peaks* were obtained by integrating over the 0.05 Å^-1^ ≤ *Q_z_* ≤ 0.75 Å^-1^ region. The *Bragg peaks* (A, B, C) were fitted using the sum of Voigt profiles (solid line) and de-convoluted into separate peaks (dashed lines) corresponding to the two {1,0}+{0,1} and {1,-1} Bragg peaks. A’, B’, and C” show the sum of the two DMPG {1,0}+{0,1} and {1,-1} *Bragg rods* at times *t*_1_ and *t*_2_ after the injection of hTau40. The *Bragg rods* were obtained by integrating over the 1.35 Å^-1^ ≤ *Q*_xy_ ≤ 1.55 Å^-1^ region and fitted (solid line) by approximating the coherently scattering part of the alkyl tail by a cylinder of constant electron density. Each of the {0,1}, {1,0} and {-1,1} *Bragg rods* are shown as dashed lines in bottom row. The *Bragg peaks* and *rods* associated with the tau protein is indicated by red asterisks *. The protein peak *Bragg rod* (C’) were obtained by integrating over the 1.25 Å^-1^ ≤ *Q*_xy_ ≤ 1.35 Å^-1^ region.

**Figure S2**: *Bragg peaks* (top row) and *rods* (bottom row) of a DMPG monolayer at the air/water interface at 25 mN/m and 25°C before (A, A’) and *t*_1_ = 2.5 hr (B, B’) and *t*_2_ = 12 hr (C, C’ and C”) after the injection of K18. *Bragg peaks* were obtained by integrating over the 0.05 Å^-1^ ≤ *Q_z_* ≤ 0.75 Å^-1^ region. The *Bragg peaks* were fitted using the sum of two Voigt profiles (solid line) and de-convoluted into separate peaks (dashed lines) corresponding to the two {1,0}+{0,1} and {1,-1} Bragg peaks. A’, B”, and C” show the sum of the two DMPG {1,0}+{0,1} and {1,-1} *Bragg rods* *t*_1_ and *t*_2_ after the injection of K18. The *Bragg rods* were obtained by integrating over the 1.35 Å^-1^ ≤ *Q*_xy_ ≤ 1.55 Å^-1^ region and fitted (solid line) by approximating the coherently scattering part of the alkyl tail by a cylinder of constant electron density. Each of the {0,1}, {1,0} and {-1,1} *Bragg rods* are shown as dashed lines in bottom row. The *Bragg peaks* and *rods* associated with the tau protein is indicated by *. The protein peak *Bragg rod* (B’ and C’) were obtained by integrating over the 1.25 Å^-1^ ≤ *Q*_xy_ ≤ 1.35 Å^-1^ region.

**Figure S3**: *Bragg peaks* (top row) and *rods* (bottom row) of a DMPG monolayer at the air/water interface at 25 mN/m and 25°C before (A, A’) and *t*_1_ = 2.5 hr (B, B’) and *t*_2_ = 12 hr (C, C’ and C”) after the injection of hTau40/3Epi. *Bragg peaks* were obtained by integrating over the 0.05 Å^-1^ ≤ *Q_z_* ≤ 0.75 Å^-1^ region. The *Bragg peaks* were fitted using the sum of two Voigt profiles (solid line) and de-convoluted into separate peaks (dashed lines) corresponding to the two {1,0}+{0,1} and {1,-1} Bragg peaks. A’, B”, and C” show the sum of the two DMPG {1,0}+{0,1} and {1,-1} *Bragg rods* *t*_1_ and *t*_2_ after the injection of hTau40/3Epi. The *Bragg rods* were obtained by integrating over the 1.35 Å^-1^ ≤ *Q*_xy_ ≤ 1.55 Å^-1^ region and fitted (solid line) by approximating the coherently scattering part of the alkyl tail by a cylinder of constant electron density. Each of the {0,1}, {1,0} and {-1,1} *Bragg rods* are shown as dashed lines in bottom row. The *Bragg peaks* and *rods* associated with the tau protein is indicated by red asterisks *. The protein peak *Bragg rod* (B’ and C’) were obtained by integrating over the 1.25 Å^-1^ ≤ *Q*_xy_ ≤ 1.35 Å^-1^ region.

**Table S1**. Structural parameters obtained from GIXD measurements of the full-length wildtype hTau40:DMPG film at the air/water interface at 25ºC before (DMPG) and at two time points (*t*_1_ = 2.5 hrs and *t*_2_  = 12 hrs) after the injection of hTau40.

**Table S1A**: Structural parameters extracted from the DMPG diffraction peaks.

| **Sample**  (Surface Pressure) | **Distorted hexagonal unit cell dimensions**  ***a***, ***b***, ***γ***  (Å, Å, degrees) | | | **Area per lipid**  **molecule**  (Å^2^) | **Integrated**  **intensity**  (%) | **Coherence**  **length**  *L*_c_  (Å) | **Tilt angle**  *t*  (°) | **Tilt dir.**  **from NN, non-symmetry**  (°) | ***σ***  (Å) |
| --- | --- | --- | --- | --- | --- | --- | --- | --- | --- |
| DMPG  (25 mN/m) | 4.93  ± 0.01 | 4.93  ± 0.01 | 118.8  ± 0.4 | 42.6  ± 0.1 | 46073  (100) | 14.0  ± 0.5 | 18.1  ± 1.0 | 0 | 0.5  ± 0.2 |
| hTau40:DMPG  *t*_1_ = 2.5 hrs  (34 mN/m) | 4.92  ± 0.01 | 4.92  ± 0.01 | 119.0  ± 0.4 | 42.4  ± 0.2 | 41697  (90) | 11.5  ± 0.5 | 15.5  ± 1.0 | 0 | 0.6  ± 0.2 |
| hTau40:DMPG  *t*_2_ = 12 hrs  (39 mN/m) | 4.96  ± 0.01 | 4.96  ± 0.01 | 118.0  ± 0.4 | 43.4  ± 0.1 | 6143  (13) | 11.7  ± 0.5 | 16.7  ± 1.0 | 0 | 0.6  ± 0.2 |

Due to the constant area of the trough the protein insertion into the lipid monolayer following its injection caused the surface pressure of the protein/lipid film to increase during the experiment.

*L_c_* is the length of the coherently scattering part of the alkyl tail measured along its backbone.

Tilt angle is measured from the surface normal. The tilt angle is measured between the direction of nearest neighbor and the projection of the alkyl tail on the subphase surface. Nearest neighbor (NN) is along ***a*** + ***b*,** where ***a*** and ***b*** are the 2D unit cell vectors**.** *σ* is the Debye-Waller factor or root mean-square molecular displacement.

**Table S1B**: In-plane coherence length (*L_xy_*) values of the DMPG diffraction peaks.

| **Sample**  (Surface Pressure) | **In-plane Bragg peaks**  **coherence length**  *L_xy_* (Å) ± 10 Å | |
| --- | --- | --- |
|  | ***L*_10+01_** | ***L*_1-1_** |
| DMPG | 170 | 310 |
| hTau40:DMPG *t*_1_ = 2.5 hrs | 90 | 500 |
| hTau40:DMPG *t*_2_ = 12 hrs | 100 | 240 |

*L_xy_* is the in-plane coherence length, an average length over which the scattering units are in registry.

**Table S1C**: Structural parameters extracted from the hTau40 protein peak at *Q_xy_*=1.34Å^-1^.

| **Sample**  (Surface Pressure) | ***d*-spacing**  (Å) | **Integrated**  **intensity**  (%) | **Coherence**  **length**  *L*_c_ (Å) | **Tilt angle**  *t* (°) | ***σ***  (Å) | **In-plane Bragg peaks**  **coherence length**  *L_xy_* (Å) |
| --- | --- | --- | --- | --- | --- | --- |
| hTau40  *t*_1_ = 2.5 hrs | - | - | - | - | - | - |
| hTau40  *t*_2_ = 12 hrs | 4.74 ± 0.02 | 1397  (100) | 7.2 ± 1 | 0 | 0.83 ± 0.2 | 200 ± 10 Å |

*L_c_* is the length of the coherently scattering part of the protein.

*L_xy_* is the in-plane coherence length, an average length over which the scattering units are in registry.

*σ* is the Debye-Waller factor or root mean-square molecular displacement.

**Table S2**. Structural parameters obtained from GIXD measurements of truncated tau construct K18:DMPG film at the air/water interface at 25ºC before (DMPG) and at two time points after (*t*_1_ = 2.5 hr and *t*_2_ ~12 hr) the injection of K18.

**Table S2A**: Structural parameters extracted from the DMPG diffraction peaks.

| **Sample**  (Surface Pressure) | **Distorted hexagonal unit cell dimensions**  ***a***, ***b***, ***γ***  (Å, Å, degrees) | | | **Area per lipid**  **molecule**  (Å^2^) | **Integrated**  **intensity**  (%) | **Coherence**  **length**  *L*_c_  (Å) | **Tilt angle**  *t*  (°) | **Tilt dir.**  **from NN, non-symmetry**  (°) | ***σ***  (Å) |
| --- | --- | --- | --- | --- | --- | --- | --- | --- | --- |
| DMPG  (25 mN/m) | 4.93  ± 0.01 | 4.93  ± 0.01 | 118.8  ± 0.4 | 42.6  ± 0.1 | 46073  (100) | 14.0  ± 0.5 | 18.1  ± 1.0 | 0 | 0.5  ± 0.2 |
| K18:DMPG  *t*_1_ = 2.5 hrs  (45 mN/m) | 4.96  ± 0.01 | 4.96  ± 0.01 | 118.1  ± 0.4 | 43.4  ± 0.2 | 32586  (70) | 11.6  ± 0.5 | 19.5  ± 1.0 | 0 | 0.5  ± 0.2 |
| K18:DMPG  *t*_2_ = 12 hrs  (46 mN/m) | 5.00  ± 0.01 | 5.00  ± 0.01 | 120 | 43.2  ± 0.2 | 14327  (30) | 10.5  ± 0.5 | 13.6  ± 1.0 | 0 | 0.6  ± 0.2 |

Due to the constant area of the trough the protein insertion into the lipid monolayer following its injection caused the surface pressure of the protein/lipid film to increase during the experiment.

*L_c_* is the length of the coherently scattering part of the alkyl tail measured along its backbone.

Tilt angle is measured from the surface normal. The tilt angle is measured between the direction of nearest neighbor and the projection of the alkyl tail on the subphase surface. Nearest neighbor (NN) is along ***a*** + ***b*,** where ***a*** and ***b*** are the 2D unit cell vectors**.** *σ* is the Debye-Waller factor or root mean-square molecular displacement.

**Table S2B**: In-plane coherence length (*L_xy_*) values of the DMPG diffraction peaks.

| **Sample**  (Surface Pressure) | **In-plane Bragg peaks**  **coherence length**  *L_xy_* (Å) ± 10 Å | |
| --- | --- | --- |
|  | ***L*_10+01_** | ***L*_1-1_** |
| DMPG | 170 | 310 |
| K18:DMPG, *t*_1_ = 2.5 hrs | 100 | 200 |
| K18:DMPG, *t*_2_ = 12 hrs | 40 | 40 |

*L_xy_* is the in-plane coherence length, an average length over which the scattering units are in registry.

**Table S2C**: Structural parameters extracted from the K18 protein peak at *Q_xy_*=1.34Å^-1^.

| **Sample**  (Surface Pressure) | ***d*-spacing**  (Å) | **Integrated**  **intensity**  (%) | **Coherence**  **length**  *L*_c_ (Å) | **Tilt angle**  *t* (°) | ***σ***  (Å) | **In-plane Bragg peaks**  **coherence length**  *L_xy_* (Å) |
| --- | --- | --- | --- | --- | --- | --- |
| K18  *t*_1_ = 2.5 hrs | 4.74 ± 0.03 | 2415  (56) | 4.5* | 0 | 0.6 ± 0.2 | 150 ± 20 Å |
| K18  *t_2_* = 12 hrs | 4.77 ± 0.02 | 4272  (100) | 6.5 ± 1 | 0 | 0.7 ± 0.2 | 120 ± 10 Å |

*L_c_* is the length of the coherently scattering part of the protein.

*L_xy_* is the in-plane coherence length, an average length over which the scattering units are in registry.

*σ* is the Debye-Waller factor or root mean-square molecular displacement.

* Due to weak scattering and high background, the extraction of the *Bragg rod* and resulting structural data was with a higher degree of uncertainty.

**Table S3**. Structural parameters obtained from GIXD measurements of the hyperp-hosphorylation mimic mutant hTau40/3Epi:DMPG film at the air/water interface at 25ºC before (DMPG) and at two time points after (*t*_1_ = 2.5 hr and *t*_2_ ~12 ~~(8.5?)~~ hr) the injection of hTau40/3EPi.

**Table S3A**: Structural parameters extracted from the DMPG diffraction peaks.

| **Sample**  (Surface Pressure) | **Distorted hexagonal unit cell dimensions**  ***a***, ***b***, ***γ***  (Å, Å, degrees) | | | **Area per lipid**  **molecule**  (Å^2^) | **Integrated**  **intensity**  (%) | **Coherence**  **length**  *L*_c_  (Å) | **Tilt angle**  *t*  (°) | **Tilt dir.**  **from NN, non-symmetry** (°) | ***σ***  (Å) |
| --- | --- | --- | --- | --- | --- | --- | --- | --- | --- |
| DMPG  (25 mN/m) | 4.93  ± 0.01 | 4.93  ± 0.01 | 118.8  ± 0.4 | 42.6  ± 0.1 | 46073  (100) | 14.0  ± 0.5 | 18.1  ± 1.0 | 0 | 0.5  ± 0.2 |
| hTau40/3Epi:  DMPG  *t*_1_ = 2.5 hrs  (47 mN/m) | 4.95  ± 0.01 | 4.95  ± 0.01 | 118.2  ± 0.4 | 43.2  ± 0.1 | 37070  (80) | 10.9  ± 0.5 | 18.0  ± 1.0 | 0 | 0.6  ± 0.1 |
| hTau40/3Epi:  DMPG  *t*_2_ = 12 hrs  (44 mN/m) | 4.98  ± 0.01 | 4.98  ± 0.01 | 117.6  ± 0.4 | 43.9  ± 0.1 | 26549  (58) | 10.9  ± 0.5 | 19.2  ± 1.0 | 0 | 0.6  ± 0.2 |

Due to the constant area of the trough the protein insertion into the lipid monolayer following its injection caused the surface pressure of the protein/lipid film to increase during the experiment.

*L_c_* is the length of the coherently scattering part of the alkyl tail measured along its backbone.

Tilt angle is measured from the surface normal. The tilt angle is measured between the direction of nearest neighbor and the projection of the alkyl tail on the subphase surface. Nearest neighbor (NN) is along ***a*** + ***b*,** where ***a*** and ***b*** are the 2D unit cell vectors**.** *σ* is the Debye-Waller factor or root mean-square molecular displacement.

**Table S3B**: In-plane coherence length (*L_xy_*) values of the DMPG diffraction peaks.

| **Sample**  (Surface Pressure) | **In-plane Bragg peaks**  **coherence length**  *L_xy_* (Å) ± 10 Å | |
| --- | --- | --- |
|  | ***L*_10+01_** | ***L*_1-1_** |
| DMPG | 170 | 310 |
| hTau40/3Epi:DMPG, *t*_1_ = 2.5 hrs | 80 | 170 |
| hTau40/3Epi:DMPG, *t*_2_ = 12 hrs | 70 | 110 |

*L_xy_* is the in-plane coherence length, an average length over which the scattering units are in registry.

**Table S3C**: Structural parameters extracted from the hTau40/3Epi protein peak at *Q_xy_*=1.34Å^-1^.

| **Sample**  (Surface Pressure) | ***d*-spacing**  (Å) | **Integrated**  **intensity**  (%) | **Coherence**  **length**  *L*_c_ (Å) | **Tilt angle**  *t* (°) | ***σ***  (Å) | **In-plane Bragg peaks**  **coherence length**  *L_xy_* (Å) |
| --- | --- | --- | --- | --- | --- | --- |
| hTau40/3Epi  *t*_1_ = 2.5 hrs | 4.72  ± 0.02 | 1789  (37) | 5.5  ± 1 | 0 | 0.63  ± 0.2 | 110 |
| hTau40/3Epi  *t_2_* = 12 hrs | 4.72  ± 0.02 | 4795  (100) | 5.5  ± 1 | 0 | 0.67  ± 0.2 | 130 |

*L_c_* is the length of the coherently scattering part of the protein.

*L_xy_* is the in-plane coherence length, an average length over which the scattering units are in registry.

*σ* is the Debye-Waller factor or root mean-square molecular displacement.

**Figure S4**: Grazing incidence X-ray diffraction results of hTau40 (A), K18 (B) and hTau40/3Epi (C) adsorbed to the air/water interface at 25°C. The scattering background is not subtracted from the data. As shown, no *Bragg peaks* were observed. This indicates that the proteins adsorbed at the air/water interface did not form repeating structural features, such as diffraction peak at *Q*_xy_ = 1.34 Å^-1^ that corresponds to β-sheet ordering. Such ordering was observed when the same tau proteins were associated with anionic lipid membranes at the air/water interface. For the hTau40/3Epi results (C), the scattering signal above *Q*_xy_ 1.5 Å^-1^, which is much larger than the region where β-sheet diffraction signal is expected, is noisy. The spikes of intensity were uniformly distributed along the *Q*_z_ direction (direction perpendicular to the air/water interface) and therefore did not correspond to any structural features.


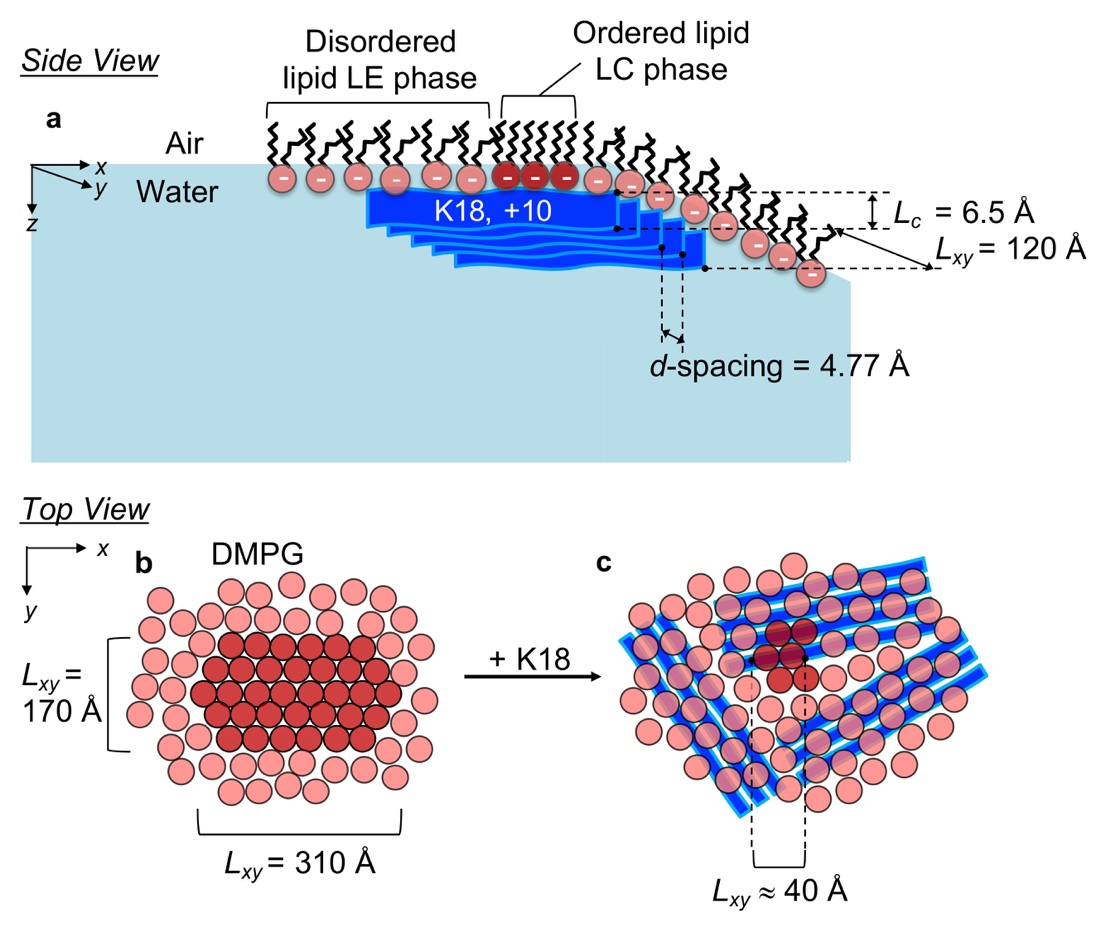


**Figure S5**: Schematics of β-sheet enriched K18 assemblies at the anionic DMPG monolayer surface. **a**: side view (parallel or in-plane to the lipid membrane) shows an ordered K18 assembly associated with the anionic DMPG monolayer at the air/water interface. *d*-spacing and coherence lengths (*L_xy_*) shown are for the K18 oligomer. *L_c_* is the out-of-plane length of the coherently scattering moiety of the K18 assembly. **b**: top views (perpendicular or out-of-plane to the lipid membrane) of the membrane before (**b**) and after (**c**) K18 association. The *L_xy_* values in these schematics are those of the ordered lipid domains. After 12 hrs the average size of the ordered DMPG domains deceased from about 300 Å to 40 Å (Table S2B). The orientation of the K18 β-sheet arrangements vis-à-vis the orientation of the DMPG lattice cannot be determined from the experimental data.


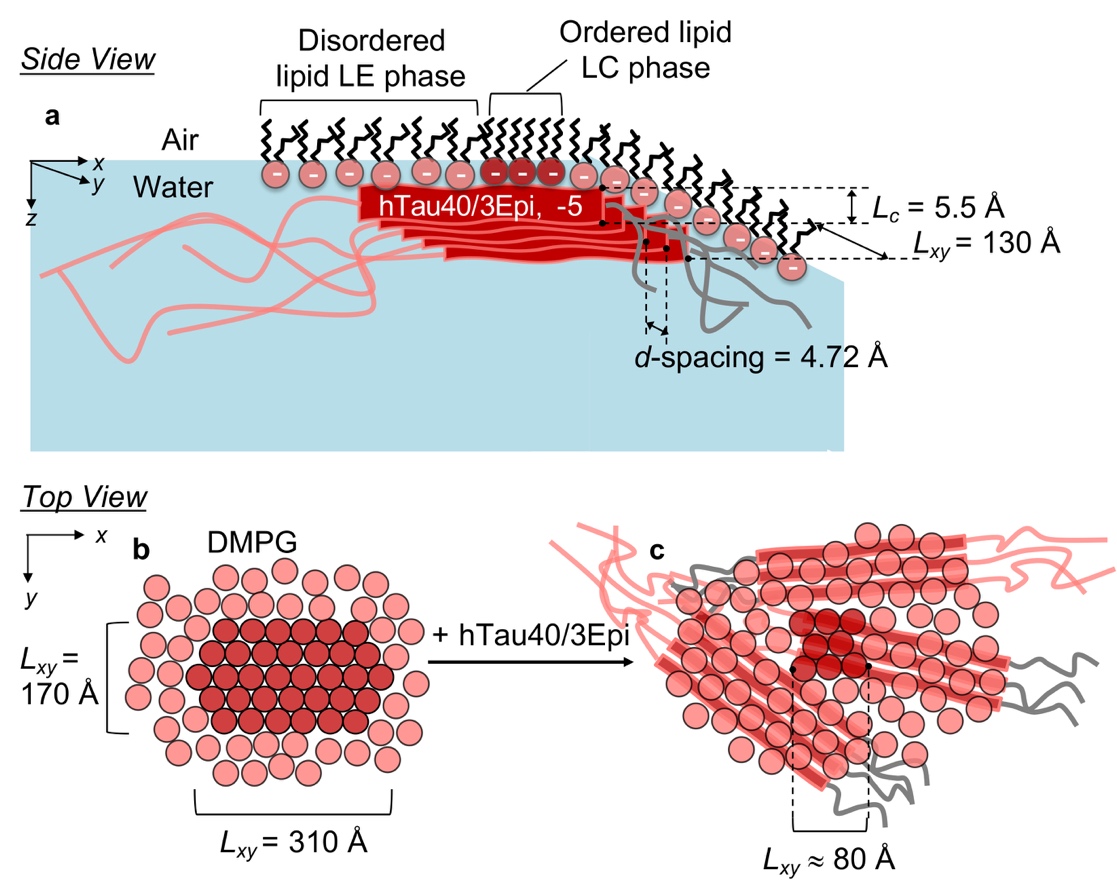


**Figure S6**: Schematics of β-sheet enriched hTau40/3Epi assemblies at the anionic DMPG monolayer surface. **a**: side view (parallel or in-plane to the lipid membrane) shows an ordered hTau40/3Epi assembly associated with the anionic DMPG monolayer at the air/water interface. *d*-spacing and coherence lengths (*L_xy_*) shown are for the hTau40/3Epi oligomer. *L_c_* is the out-of-plane length of the coherently scattering moiety of the hTau40/3Epi assembly. **b**. top views (perpendicular or out-of-plane to the lipid membrane) of the membrane before (left) and after (right) hTau40/3Epi association. The *L_xy_* values in these schematics are those of the ordered lipid domains. After 12 hrs the average size of the ordered DMPG domains deceased from about 300 Å to 80 Å (Table S3B). The orientation of the hTau40/3Epi β-sheet arrangements vis-à-vis the orientation of the DMPG lattice cannot be determined from the experimental data.

**Video S1**: 1 μM hTau40 incubated for approximately 12 hours with a DMPG monolayers held at 25 mN/m on water subphase at 25°C. In the video, the platinum Wilhelmy plate is seen moving around and in and out of the water subphase containing the lipid-tau protein film at the air/water interface. Note that the tau-DMPG film appears viscous and can be dragged up with the Wilhelmy plate as it is pulled up from the film at the air/water interface. Video file is uploaded with manuscript submission as a separate file.

**Video S2**: 0.25 μM amyloid-β peptide (Aβ40) incubated for approximately 12 hrs with a DMPG monolayers held at 25 mN/m on water subphase at 25°C. In the video, the platinum Wilhelmy plate is seen moving around and in and out of the water subphase containing the lipid-Aβ protein film at the air/water interface. Notice that the lipid-Aβ film breaks away easily from the Wilhelmy plate as it is pulled up from the film at the air/water interface. Video file is uploaded with manuscript submission as a separate file.
